# Serum 25-hydroxyvitamin D_3_ and 24*R*,25-dihydroxyvitamin D_3_ concentrations in adult dogs are more substantially increased by oral supplementation of 25-hydroxyvitamin D_3_ than by vitamin D_3_

**DOI:** 10.1017/jns.2017.8

**Published:** 2017-06-20

**Authors:** Lauren R. Young, Robert C. Backus

**Affiliations:** Department of Veterinary Medicine and Surgery, College of Veterinary Medicine, University of Missouri, Columbia, MO 65211, USA

**Keywords:** Cholecalciferol, 25-Hydroxycholecalciferol, 24*R*,25-dihydroxycholecalciferol, Supplementation in dogs, 24*R*,25(OH)_2_D_3_, 24*R*,25-dihydroxyvitamin D_3_, 25(OH)D_3_, 25-hydroxyvitamin D_3_, BW, body weight, D_3_, vitamin D_3_

## Abstract

We previously found a weak response in serum 25-hydroxyvitamin D_3_ (25(OH)D_3_) concentrations when dogs were supplemented with oral vitamin D_3_ (D_3_). In the present study, we determined the relative potency of oral 25(OH)D_3_ compared with D_3_ for increasing vitamin D status in dogs with low serum 25(OH)D concentrations. Four male and three female, 4-year-old, intact, lean, genetically related, Chinese-crested/beagle dogs were studied in a randomised, single cross-over trial. After feeding a low-vitamin D diet (<4 IU/100 g) for 30 d, four dogs received daily D_3_ supplementation at 2·3 µg/kg body weight^0·75^, while three dogs received a molar equivalency as 25(OH)D_3_. The supplements, dissolved in ethanol, were applied to a commercial treat for consumption. Serum 25(OH)D_3_ and 24*R*,25-dihydroxyvitamin D_3_ (24*R*,25(OH)_2_D_3_) were analysed weekly using a validated HPLC method. Both supplementations increased (*P* ≤ 0·01) serum 25(OH)D_3_ concentrations. However, oral 25(OH)D_3_ resulted in greater (*P* < 0·0001) concentrations than D_3_ by week 1, with a difference of 173 % (*P* < 0·0001) by week 2. The supplementation period was limited to 14 d after serum 25(OH)D_3_ concentrations were not appearing to plateau. Thereafter, a washout period of 1 month separated the cross-over. Following 25(OH)D_3_, but not D_3_ supplementation, serum 24*R*,25(OH)_2_D_3_ concentrations increased (*P* ≤ 0·02), 3 to 5 weeks after initiating supplementation. Vitamin D status, as indicated by serum 25(OH)D_3_ and 24*R*,25(OH)_2_D_3_ concentrations, is more rapidly and efficiently increased in adult dogs by oral supplementation of 25(OH)D_3_ than D_3_.

In recent years, several reports in the veterinary literature have associated low vitamin D status with many different disease processes in dogs, including chronic kidney disease^(^^1^^)^, congestive heart failure^(^^2^^)^, inflammatory bowel disease^(^^3^^)^, mast cell tumour^(^^4^^)^ and cancer^(^^5^^)^. Whether low vitamin D status is causative or a consequence of disease has not been established. Nonetheless, studies such as these have brought attention to vitamin D status and health in adult dogs.

Dogs are unable to adequately synthesise vitamin D_3_ (D_3_) in their skin in response to UV light^(^^6^^)^ and, therefore, are reliant upon their diet to supply their vitamin D needs. The dietary vitamin D requirement for adult dogs is not clearly established. The current adequate intake of vitamin D recommended by the National Research Council (NRC) for dogs in all life stages is based on findings of studies for the prevention of skeletal abnormalities in puppies^(^^7^^)^. This vitamin D recommendation, though supporting of normal bone growth and maintenance in a puppy, may not be sufficient for other health outcomes in adult dogs.

It is widely accepted that the best indicator of vitamin D status is serum 25-hydroxyvitamin D (25(OH)D), as it is the most abundant circulating metabolite of vitamin D, and its concentration is determined by vitamin D intake^(^^8^^)^. Reports of serum 25(OH)D concentrations amongst apparently healthy dogs are quite varied^(^^5^^,^^9^^–^^11^^)^. One study attempted to define vitamin D sufficiency in healthy, adult dogs by comparing the relationship between serum 25(OH)D concentrations and intact parathyroid hormone (iPTH)^(^^5^^)^. Investigators found that the median and variance in iPTH observations among dogs declined to a plateau when 25(OH)D concentrations were at 100 ng/ml. They also found a significant drop in variability of serum mean C-reactive protein concentrations, a marker of chronic inflammation, when 25(OH)D concentrations were 100–120 ng/ml. The authors concluded that vitamin D sufficiency is indicated when serum 25(OH)D is 100–120 ng/ml, and that many apparently healthy dogs are vitamin D insufficient.

The present authors found in a previous cohort survey of forty-six adult, healthy dogs^(^^11^^)^ that 71·7 % had serum 25(OH)D concentrations below 100 ng/ml. A subsequent D_3_ supplementation trial was conducted in thirteen of the dogs deemed vitamin D insufficient. Seven dogs received D_3_ in an olive oil solution on their food at 2·3 µg/kg body weight (BW)^0·75^ per d, an amount that was 5·1 times the NRC recommended allowance but not in excess of the safe upper limit for maintenance of adult dogs (2·6 µg/kg BW^0·75^)^(^^12^^)^. Six dogs received an olive oil placebo. Unexpectedly, we found that D_3_ supplementation, at an oral dosage that we believed to be substantial, did not significantly increase serum 25(OH)D concentrations above baseline. At the end of 9 to 10 weeks of supplementation, only a modest difference (12 %) in vitamin D status resulted between the treated and control dogs.

The cause of the poor response to vitamin D supplementation was not apparent, but it prompted us to investigate the use of 25(OH)D_3_ as a supplement in dogs. The objective of this study was to determine the relative potency of 25(OH)D_3_ as compared with D_3_ for increasing vitamin D status in dogs deemed vitamin D insufficient. Based on a previous report in dogs^(^^13^^)^, we hypothesised that vitamin D status, as measured by serum 25(OH)D_3_ and 24*R*,25-dihydroxyvitamin D_3_ (24*R*,25(OH)_2_D_3_) concentrations, would be significantly more responsive to 25(OH)D_3_ than D_3_.

## Materials and methods

All procedures were reviewed and approved by our institution's animal care and use committee. Four male and three female institution-owned, 4-year-old, intact, Chinese crested/beagle dogs were studied in a randomised, single cross-over trial. The dogs belonged to the same litter and were in ideal body condition (body condition score 5/9), with BW ranging from 5·9 to 10·7 kg. The dogs were consuming a commercially produced laboratory diet formulated to meet Association of American Feed Control Officials (AAFCO) dog food nutrient profiles for adult canine maintenance fed *ad libitum* (LabDiet 5006; PMI Nutrition International, Inc.). The vitamin D content of the diet as measured by an independent laboratory (Covance Laboratories, Inc.) was 330 IU/100 g.

### Study design

Upon obtaining the dogs for study, jugular venous blood was collected following an overnight food withholding, and serum was harvested for 25(OH)D analysis. Immediately prior to entry into the trial, jugular venous blood was again collected following an overnight food withholding for complete blood counts with manual differentials and clinical serum chemistry analyses to screen each dog for underlying disease. The dogs were subsequently transitioned over a 7-d period to a commercially produced diet identical in ingredients to their previous diet and produced by the same manufacturer, with the exception of no vitamin D supplementation, and maintained on the diet for the duration of the study. The vitamin D content of the diet as measured by the same laboratory was <4 IU/100 g.

Dogs were weighed weekly, fed an amount of diet to maintain ideal BW, and evaluated each week for vitamin D status throughout the study. At each evaluation, jugular venous blood was obtained following an overnight food withholding, and serum was harvested for 25(OH)D_3_ and 24*R*,25(OH)_2_D_3_ analyses. Following a 1-month run-in period consuming the diet, the dogs voluntary consumed 2–3 g of a treat (Canine Carry Out; Big Heart Pet Brands) to which small volumes (5–10 µl) of ethanolic solutions were applied of D_3_ (cholecalciferol; Sigma-Aldrich) at a dosage of 2·3 µg/kg BW^0·75^ per d (*n* 4), or a molar equivalent dosage as 25(OH)D_3_ (Sigma-Aldrich) (*n* 3). The vitamin D content of the treat measured by the same laboratory was <4 IU/100 g. Treatments were given daily until serum 25(OH)D_3_ concentrations determined on a weekly basis exceeded 100 ng/ml, at which time the washout period began. When serum 25(OH)D_3_ concentrations returned to baseline, the treatments were resumed in a cross-over assignment.

### Laboratory analyses

The clinical haematology (Sysmex xT-2000i; Sysmex America, Inc.) and serum chemistry analyses (Beckman AU 400e; Beckman Coulter, Inc.) were performed at the University of Missouri Veterinary Medical Diagnostic Laboratory, Columbia, MO. Serum concentrations of the vitamin D metabolites were determined using a modification of an HPLC method previously reported^(^^14^^)^, but with 25(OH)D_2_ in place of ^3^H-labelled 25(OH)D_3_ as internal standard.

### Statistical analysis

Statistical analyses were performed using proprietary software (SAS^®^ 9.3; SAS Institute). All variable observations were found to be normally distributed except 24*R*,25(OH)_2_D_3_ concentrations between the treatments at weeks 2 and 4. For normally distributed observations, the significance of differences within and between treatments was determined with paired *t* tests. For non-normally distributed observations, significance was tested within and between treatments with a signed rank test.

The fractional rate of decline in serum 25(OH)D_3_ concentration (*k*) was determined from the slope of linear-regressed, log-transformed, serum 25(OH)D_3_ concentrations observed during the 4 weeks following supplement withdrawal using the equation: *t*_½_ = 0·693/*k*, where 0·693 equals the natural log of 2 and *k* is the weekly fractional rate of decrease in 25(OH)D_3_ concentration. This assumes a first-order washout curve as indicated by findings in a previous study^(^^15^^)^. *P* values ≤0·05 were considered significant.

## Results

**Fig. 1.**
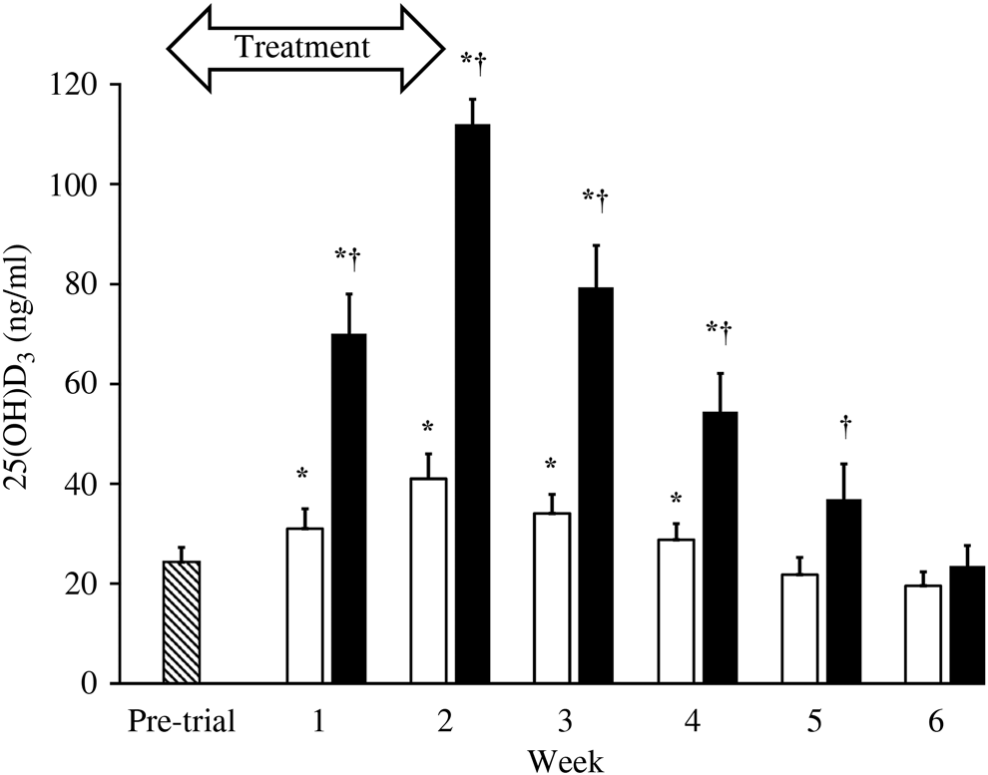
Serum 25-hydroxyvitamin D_3_ (25(OH)D_3_) concentrations prior to entry into the trial (*n* 7; 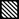) and weekly throughout vitamin D_3_ (*n* 7; □) and 25(OH)D_3_ (*n* 7; ■) supplementation and washout period. The treatment period is highlighted by the arrow. Values are means, with standard errors represented by vertical bars. * Mean value was significantly different from that at pre-trial (*P* ≤ 0·01, paired *t* test). † Mean value was significantly different from that for vitamin D_3_ supplementation (*P* ≤ 0·02, paired *t* test).

**Fig. 2.**
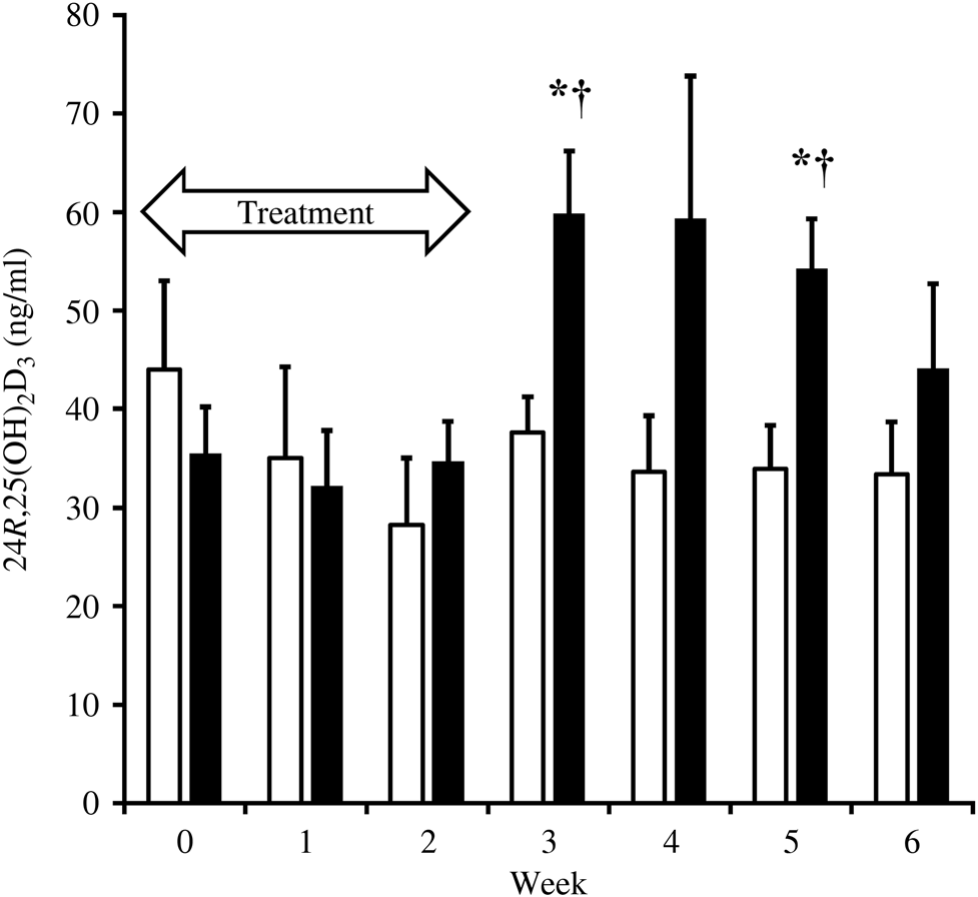
Serum concentrations of 24*R*,25-dihydroxyvitamin D_3_ (24*R*,25(OH)_2_D_3_) during supplementation of vitamin D_3_ (*n* 7; □) and 25(OH)D_3_ (*n* 7; ■) and washout period. The treatment period is highlighted by the arrow. Values are means, with standard errors represented by vertical bars. * Mean value was significantly different from that at week 0 (*P* ≤ 0·02, paired *t* test). † Mean value was significantly different from that for vitamin D_3_ supplementation (*P* ≤ 0·008, paired *t* test).

With only a few exceptions, complete blood counts and serum chemistry analyses results among the dogs were within the clinical laboratory reference ranges. The exceptions were not of parameters relevant to the study. The mean 25(OH)D_3_ concentration for all dogs immediately prior to entry into the trial was 24 (sd 10) ng/ml. At 1 week after supplementations with both D_3_ and 25(OH)D_3_, serum 25(OH)D_3_ concentrations increased significantly above baseline (*P* < 0·01, *P* < 0·0001, respectively). However, supplementation with 25(OH)D_3_ resulted in over two times greater serum 25(OH)D_3_ concentrations at week 1 (mean 70 *v*. 31 ng/ml; *P* < 0·0001). By the second week of supplementation with 25(OH)D_3_, mean 25(OH)D_3_ concentration reached 112 (sd 14) ng/ml, which was significantly greater (*P* < 0·0001) than concentrations with D_3_ supplementation (41 (sd 13) ng/ml). Supplementations during each phase of the crossover trial were discontinued after 2 weeks when mean serum 25(OH)D_3_ concentrations were found in excess of 100 ng/ml. Serum 25(OH)D_3_ concentrations declined to baseline within 4 weeks of discontinuation of supplementations. Throughout the washout period, serum 25(OH)D_3_ concentrations remained significantly greater (*P* ≤ 0·02) in dogs when they were given 25(OH)D_3_ compared with when they were given D_3_. The rate of decline in serum 25(OH)D_3_ concentration was approximately twice as rapid in dogs when given 25(OH)D_3_ (*t*½ = 1·8 weeks) than when given D_3_ (*t*½ = 3·6 weeks) (Fig. 1). Serum concentrations of 24*R*,25(OH)_2_D_3_ were significantly increased (*P* ≤ 0·02) above baseline following supplementation of 25(OH)D_3_ but not D_3_. The increase in 24*R*,25(OH)_2_D_3_ was delayed, occurring 3–5 weeks after initiation of supplementation of 25(OH)D_3_ and varied among dogs. At 3 and 5 weeks after supplementation began, serum concentrations of 24*R*,25(OH)_2_D_3_ were significantly greater (*P* ≤ 0·008) when 25(OH)D_3_ was supplemented as compared with D_3_ (Fig. 2). Peak serum 24*R*,25(OH)_2_D_3_ concentrations reached 50–119 ng/ml following treatment with 25(OH)D_3_.

## Discussion

Our objective in comparing the relative potency of 25(OH)D_3_ to D_3_ for increasing vitamin D status in dogs is based upon our previous finding of a weak response to D_3_ supplementation in dogs deemed vitamin D insufficient^(^^11^^)^. With studies indicating that dogs with chronic disease have low vitamin D concentrations^(^^1^^–^^5^^)^, as well as many healthy dogs^(^^5^^,^^9^^–^^11^^)^, research into an effective and safe means to improving vitamin D status seems warranted. The use of 25(OH)D_3_ supplementation as a means to improve vitamin D status in the dog has not been evaluated, as it has in people. Earlier work has demonstrated that 25(OH)D_3_ is absorbed in the human intestine similar to D_3_^(^^16^^)^, yet peak concentrations in serum 25(OH)D after oral administration in humans are reached much more rapidly, as compared with the slow rise in concentrations that are typically observed after D_3_ administration^(^^16^^,^^17^^)^. Recently published oral 25(OH)D_3_ supplementation studies have shown that 25(OH)D_3_ is much more efficient and rapid at increasing vitamin D status in humans than D_3_^(^^18^^–^^20^^)^.

In accordance with this, we have also found that at equivalent doses in dogs, oral supplementation with 25(OH)D_3_ is much more effective and rapid than D_3_ in raising serum 25(OH)D_3_ concentrations above a previously reported minimum indicative of vitamin D sufficiency^(^^5^^)^ (Fig. 1). While both supplementations significantly increased serum 25(OH)D_3_ concentrations above baseline, oral 25(OH)D_3_ was at least 5·2 times as potent as D_3_ after just 2 weeks of supplementation. Due to an unacceptably high trajectory of serum 25(OH)D_3_ concentrations following supplementation with 25(OH)D_3_, the trial was discontinued after 2 weeks, before equilibrium could be safely established. Therefore, it is possible that this is an underestimation of the true potency of 25(OH)D_3_ relative to D_3_.

Our present finding of a significant increase in serum 25(OH)D_3_ concentrations following supplementation with D_3_ is in contrast to our previous study results^(^^11^^)^. Although the D_3_ doses were the same (2·3 µg/kg BW^0·75^ per d), the vehicle of D_3_ supplement delivery differed. Bioavailability of D_3_ may be greater with the treat application presently used compared with our previous top-dressing of food with an olive oil solution of D_3_. However, dogs in each study willingly accepted both methods of supplement delivery. Studies on factors affecting vitamin D absorption, distribution and metabolism in dogs are lacking. Additionally, the present study should be considered to be much more controlled than our previous work, in which privately owned dogs were studied and D_3_ supplementation depended on owner compliance.

Concentration of 24*R*,25(OH)_2_D in serum is well established to positively correlate with serum 25(OH)D concentration in the dog^(^^21^^)^. Serum 24*R*,25(OH)_2_D_3_ concentrations were significantly increased by supplementation of 25(OH)D_3_ but not with D_3_. However, this does not occur until 3 and 5 weeks following initiation of supplementation. The variation in production of this metabolite amongst the dogs probably resulted in the insignificant difference in concentrations between the supplementations at the second week of the washout period (Fig. 2). This probably indicates a lag time in the 24-hydroxylase activity in the kidney necessary to convert 25(OH)D_3_ to 24*R*,25(OH)_2_D_3_, as has been demonstrated in humans^(^^22^^)^.

A noteworthy limitation of this study is the small number of dogs and lacking of investigation of factors evidenced to influence serum 25(OH)D concentrations in dogs, such as sex (males > females), reproductive status (intact > neutered) and breed^(^^10^^)^. In conclusion, our findings indicated that oral supplementation of 25(OH)D_3_ is at least 5·2 times as potent as D_3_ for increasing vitamin D status in dogs with low serum 25(OH)D concentrations. While this work is supportive of the use of 25(OH)D_3_ as a supplement means to improve vitamin D status in the dog, a safe dosage was not identified and will require further investigation.

## References

[1] Gerber B, Hassig M & Reusch CE (2003) Serum concentrations of 1,25-dihydroxycholecalciferol and 25-hydroxycholecalciferol in clinically normal dogs and dogs with acute and chronic renal failure. Am J Vet Res 64, 1161–1166. 13677396 10.2460/ajvr.2003.64.1161

[2] Kraus MS, Rassnick KM, Wakshlag JJ, (2014) Relation of vitamin D status to congestive heart failure and cardiovascular events in dogs. J Vet Intern Med 28, 109–115. 24205918 10.1111/jvim.12239 PMC4895547

[3] Gow AG, Else R, Evans H, (2011) Hypovitaminosis D in dogs with inflammatory bowel disease and hypoalbuminaemia. J Small Anim Pract 52, 411–418. 21797872 10.1111/j.1748-5827.2011.01082.x

[4] Wakshlag JJ, Rassnick KM, Malone EK, (2011) Cross-sectional study to investigate the association between vitamin D status and cutaneous mast cell tumours in Labrador retrievers. Br J Nutr 106, S60–S63. 22005438 10.1017/S000711451100211X

[5] Selting KA, Sharp CR, Ringold R, (2016) Serum 25-hydroxyvitamin D concentrations in dogs – correlation with health and cancer risk. Vet Comp Oncol 14, 295–305. 25041357 10.1111/vco.12101

[6] How KL, Hazewinkel HA & Mol JA (1994) Dietary vitamin D dependence of cat and dog due to inadequate cutaneous synthesis of vitamin D. Gen Comp Endocrinol 96, 12–18. 7843559 10.1006/gcen.1994.1154

[7] National Research Council *Ad Hoc* Committee on Dog and Cat Nutrition (2006) Vitamins In Nutrient Requirements of Dogs and Cats, pp. 193–245. Washington, DC: The National Academies Press.

[8] Stocklin E & Eggersdorfer M (2013) Vitamin D, an essential nutrient with versatile functions in nearly all organs. Int J Vitam Nutr Res 83, 92–100. 24491882 10.1024/0300-9831/a000151

[9] Fairweather AAC, Eason CT, Elder PA, (2013) Reference concentrations of cholecalciferol in animals: a basis for establishing non-target exposure. New Zeal J Zool 40, 280–289.

[10] Sharp CR, Selting KA & Ringold R (2015) The effect of diet on serum 25-hydroxyvitamin D concentrations in dogs. BMC Res Notes 8, 442. 26374201 10.1186/s13104-015-1360-0 PMC4570747

[11] Young LR & Backus RC (2016) Oral vitamin D supplementation at five times the recommended allowance marginally affects serum 25-hydroxyvitamin D concentrations in dogs. J Nutr Sci 5, e31. 27547394 10.1017/jns.2016.23 PMC4976120

[12] National Research Council *Ad Hoc* Committee on Dog and Cat Nutrition (2006) Nutrient requirements and dietary nutrient concentrations In Nutrient Requirements of Dogs and Cats, pp. 354–370. Washington, DC: The National Academies Press.

[13] Dusso A, Lopez-Hilker S, Rapp N, (1988) Extra-renal production of calcitriol in chronic renal failure. Kidney Int 34, 368–375. 3172645 10.1038/ki.1988.190

[14] Lensmeyer GL, Wiebe DA, Binkley N, (2006) HPLC method for 25-hydroxyvitamin D measurement: comparison with contemporary assays. Clin Chem 52, 1120–1126. 16574756 10.1373/clinchem.2005.064956

[15] Dougherty SA, Center SA & Dzanis DA (1990) Salmon calcitonin as adjunct treatment for vitamin D toxicosis in a dog. J Am Vet Med Assoc 196, 1269–1272. 2158960

[16] Stamp TC (1974) Intestinal absorption of 25-hydroxycholecalciferol. Lancet ii, 121–123. 10.1016/s0140-6736(74)91553-0 4135557

[17] Haddad JG Jr & Rojanasathit S (1976) Acute administration of 25-hydroxycholecalciferol in man. J Clin Endocrinol Metab 42, 284–290. 177440 10.1210/jcem-42-2-284

[18] Cashman KD, Seamans KM, Lucey AJ, (2012) Relative effectiveness of oral 25-hydroxyvitamin D_3_ and vitamin D_3_ in raising wintertime serum 25-hydroxyvitamin D in older adults. Am J Clin Nutr 95, 1350–1356. 22552038 10.3945/ajcn.111.031427

[19] Bischoff-Ferrari HA, Dawson-Hughes B, Stocklin E, (2012) Oral supplementation with 25(OH)D_3_ *versus* vitamin D_3_: effects on 25(OH)D levels, lower extremity function, blood pressure, and markers of innate immunity. J Bone Miner Res 7, 160–169. 10.1002/jbmr.551 22028071

[20] Jetter A, Egli A, Dawson-Hughes B, (2014) Pharmacokinetics of oral vitamin D_3_ and calcifediol. Bone 59, 14–19. 24516879

[21] Tryfonidou MA, Oosterlaken-Dijksterhuis MA, Mol JA, (2003) 24-Hydroxylase: potential key regulator in hypervitaminosis D_3_ in growing dogs. Am J Physiol Endocrinol Metab 284, E505–E513. 12441310 10.1152/ajpendo.00236.2002

[22] Wagner D, Hanwell HE, Schnabl K, (2011) The ratio of serum 24,25-dihydroxyvitamin D_3_ to 25-hydroxyvitamin D_3_ is predictive of 25-hydroxyvitamin D_3_ response to vitamin D_3_ supplementation. J Steroid Biochem Mol Biol 126, 72–77. 21605672 10.1016/j.jsbmb.2011.05.003

